# The unprotectables: A critical discourse analysis of older people’s portrayal in UK newspaper coverage of Covid-19

**DOI:** 10.1177/13675494231185539

**Published:** 2023-07-25

**Authors:** Shir Shimoni

**Affiliations:** King’s College London, UK

**Keywords:** Ageism, Covid-19, cultural representation, neoliberalism, risk

## Abstract

In this article, I draw on the systematic, policy-led negligence with which older people in the United Kingdom were handled during the first wave of the Covid-19 pandemic, as I examine their simultaneous cultural representation across four major UK newspapers. Using content and critical discourse analysis, I demonstrate that while older people were depicted mostly through the notion of their increased risk to contract and die from the virus, this risk was consistently framed as unmanageable. I adopt a Foucauldian governmentality perspective as I argue that by framing dangers as exceeding the possibility of control and insurance, the discourse of unmanageable risk helped to dismantle the protection of older people from the virus. Moreover, I demonstrate that the unmanageable risk discourse spawned a particular kind of an older subject, one who not only is unprotectable but also invisible. I discuss how older people’s invisibility – evident in the absence of their names, voices and testimonies – operated in tandem with their unprotectability, to render them palatably disposable.

## Introduction

The disproportionally high death toll of older people from Covid-19 in the United Kingdom, constituting 89.3 percent of the deaths during the first wave of the pandemic (Office for National Statistics, 2020) was anticipated by the government even before deaths began to occur. In the first action plan for the pandemic published on 3 March, the government announced that ‘there could well be an increase in deaths arising from the outbreak, particularly amongst vulnerable and elderly groups. The UK Government and Devolved Administrations will provide advice for local authorities on dealing with this challenge’ (Department of Health and Social Care, 2020). While it is not clear what ‘dealing with this challenge’ meant in the announcement, averting the risk of older people to contract and die from the virus was never pronounced as an objective. More often, what was averted through diverse policy measures was the duty to manage the risk in relation to this population.

Indeed, on 13 March, people aged 70 and over were advised to self-isolate for an unknown period, as part of the government’s ‘herd immunity’ plan (Ellyatt, 2021). Moreover, in the Coronavirus Act announced on 25 March, local authorities – responsible for the provision of elder care – were instructed to reduce their duties of addressing care needs (Daly, 2020: 988). At the same time as social care services were cut down, as sociologist Mary Daly (2020) depicts, care homes were treated by the government mainly as spaces to which older patients would and indeed have been discharged to free up National Health Service (NHS) capacity (Daly, 2020). The mission to release the NHS from the burden of treating older people was a concerted effort, noted not only in the instruction given on 2 April by the Department of Health and Social Care to care homes to admit patients discharged from hospitals without their first undergoing a Covid test (Booth, 2020), but also in the dissemination of blanket ‘Do Not Resuscitate’ orders to older patients through general practitioner (GP) surgeries at the end of March (Besley, 2020). Likewise, in mid-April, a score tool for the NHS was consolidated to prioritise intensive care unit beds on the basis of age rather than clinical need (Walker, 2020).

In this article, I draw on the systematic negligence with which the older population was handled in the United Kingdom during the outbreak of Covid-19, as I analyse the simultaneous cultural construction of older people as prone to illness and death across four UK major newspapers. By examining their media portrayal in newspaper articles that were published during the first wave of the pandemic, I demonstrate that while older people were represented primarily in relation to their risk of becoming ill and dying from Covid-19, this risk was consistently framed as unmanageable.

I adopt a Foucauldian governmentality approach and view ‘risk’ as a mechanism of governance used to manage and regulate populations (Dean, 1998; Ewald, 1991; Rose, 1999). Foucault (1991: 93–94) has famously explained governmentality as a form of political power that operates by shaping individuals’ relation to things that comprise their very being, a relation that guides their conduct to materialise in specific ways. Unmanageable risk, I propose, constitutes a discursive mechanism of governance through which a particular relation to Covid-19, risk, and older people had been produced. By focusing on the tropes of this mechanism as they were produced and disseminated across mainstream media, I explain the constitutive role of unmanageable risk in constructing older people as unprotectable.

Moreover, by constituting older people as predominantly prone to illness and death, their image as unprotectable was aligned with neoliberal policies depriving the older population from care during the crisis. While policies reducing adult social care have been in place for decades (see below), I point to the ways through which the discourse of unmanageable risk has reinforced these policies during the pandemic.

My argument in this article is threefold. First, I contend that by defining dangers as exceeding the possibility of control and insurance, the discourse of unmanageable risk was utilised to dismantle the protection of older people from the virus before, during and after massive deaths of this demographic have occurred. Second, I argue that the discourse of unmanageable risk spawns a particular kind of an older subject, one who is both unprotectable and invisible. My analysis demonstrates that the construal of older people as unprotectable operated through their invisibilisation in the media. Finally, I propose that by reconstituting them as a group of invisible, voiceless, and unprotectable people, the discourse of unmanageable risk ultimately helped to render their disposability palatable.

I begin by drawing on the entanglement of ageism and neoliberalism as the backdrop against which the discourse of unmanageable risk had operated. I then outline the methodological process of this research leading me to focus on risk as the dominant theme within the media portrayal of older people. Next, I identify and analyse three dominant discursive structures that have appeared in articles, through which risk was constituted as unmanageable in relation to older people. I begin with the binary of young versus old emerging in the beginning of the outbreak, before turning to analyse the media’s subsequent heroising of volunteers addressing the needs of older people. Finally, I discuss the ambivalent affective register of urgency and predetermination within the coverage of the care homes debacle.

## Ageism and neoliberalism

While the Covid-19 virus may be novel, the carelessness with which the UK government handled its spread among the older population is historical and follows decades of ongoing neglect of this demographic. This neglect, moreover, has been facilitated through neoliberal measures. Since the 1970s, consecutive governments have actively promoted the creation of a private care market, bringing the UK’s adult social care system today to be the most privatised one among the industrialised countries (see Brennan et al., 2012; Harrington et al., 2019). This process was enabled by more than mere reduced state investment in welfare infrastructures and the outsourcing of services to the private sector. Rather, through the adoption and implementation of neoliberal paradigms, such as the New Public Management (Farris and Marchetti, 2017; Harrington et al., 2019) by policy makers since the 1990s, adult social care in the United Kingdom has transformed into a business industry that is handled through practices of managerialisation and marketisation, with a focus on results and competition rather than accountability and quality of care (Farris and Marchetti, 2017: 115). Today, a considerable part of this industry is owned by private equities (Hudson, 2016) whose main interest in the sector has been its ability to generate a reliable cash flow (Burns et al., 2016: 4).

The backdrop of continued and intensified privatisation of adult social care in the United Kingdom – marked by the high turnover of care workers due to inadequate working conditions, the lack of proper regulation of care homes and the high rates of older people living in poverty and isolation – is crucial for understanding that the organised disregard of the older population during the pandemic stems from a wider neoliberal policy targeting this demographic for decades.

Yet, I suggest that the neglect of older people during the pandemic results not only from a continued shrinking of adult social care by neoliberal policy, but also from a deep-seated cultural ageism. Several scholars have mapped out the prevalence of ageist claims during the pandemic, noted in the portrayal of older people as a separate, homogeneous, frail, passive and dependant group (Ayalon, 2020; Schrage-Frueh and Tracy, 2020) that poses a threat to young people (Ayalon, 2020; Zhang and Liu, 2021) as well as to the health system and the economy (Cook et al., 2021; Zhang and Liu, 2021). Studies have also highlighted how ageist framings of older people in the wake of Covid-19 intensified intergenerational animosity, while deepening the social exclusion of older people (Cook et al., 2021; Zhang and Liu, 2021).

I suggest, however, that while similar ageist claims have already been documented in other contexts (Carney and Nash, 2020; see also Segal, 2013), much less thought had been dedicated to understanding how these claims are reproduced and buttressed by neoliberal policies. At the same time, research on the privatisation of adult social care has typically focused on institutional, economic and policy aspects, while overlooking the ideological role of cultural ageism in facilitating this process.

This article sets out to address some of these gaps by analysing the discourse of unmanageable risk and the entanglement of ageism and neoliberalism underpinning it. The next sections demonstrate that while the category of unmanageable risk worked to dismantle the protection of older people from the virus, it utilised the ageist conflation of older people with decline and death (see Gullette, 2004) in various ways, to justify the disposal of those who do not actively support the neoliberal project.

## Methodology

To trace the way older people were represented in mainstream media during the pandemic, I conducted a content analysis of four of the most circulated UK newspapers, using the ProQuest database. Beyond the reason of accessibility, this database was chosen because it enabled me to extract a comprehensive amount of data, and to detect trends, both major and nuanced, within the data. I focused on the following newspapers, in their digital editions (this is due to the higher numbers of reach of digital editions in comparison to print editions); two broadsheet newspapers: the *Guardian* and the *Telegraph*, and two tabloids: *Daily Mail* and the *Daily Mirror*.^1^ These newspapers differ not only in their publication type, but also in their political orientation as well as in their target audience. While the *Telegraph* and *Daily Mail* are considered right-leaning newspapers, and the *Guardian* and the *Daily Mirror* left-leaning, the *Telegraph* and the *Guardian* are thought to target upper- and middle-class audience, and the *Daily Mirror* and *Daily Mail* are targeted at lower-, middle-, and working-class audience.

Using the search terms ‘elderly’ and ‘older people’, I ran a search of articles that had appeared during each week between 3 February 2020 (when the first article featuring older people and Covid-19 was published) and 2 August 2020 (after 2 months during which such articles stopped being published). After the search yielded 3035 newspaper articles, for specificity, I decided to add the following search terms: ‘over-70s’, ‘covid’ and ‘corona’, and this yielded a number of 2185 newspaper articles (61% published by the *Telegraph*, 17% published by the *Guardian*, 15% published by the *Daily Mail* and 7% published by the *Daily Mirror*). After removing duplicate articles, the total number of articles decreased to 1242. To conduct an in-depth discourse analysis, I created a sample of articles using systematic random sampling and selected every tenth article from the total cohort. This resulted in the creation of the final sample of 124 items.

In the next stage, I utilised an inductive analysis strategy (Patton, 2002: 56–57, 453) while using NVivo software to identify emergent themes and patterns within the data. Through careful and iterated reading of each article, I formulated a set of categories depicting the various contexts within which the terms ‘older people’ and ‘elderly’ were employed in the articles. In categorising the articles, I paid attention to the main subject and thematic structure of each article, and the broader context within which the main subject is discussed. Articles were categorised into seven categories: (1) risk (51%) – older people explicitly discussed with regards to virus-related risks, (2) commentary on policies (10%) – the implications of current policies to members of society, including older people, (3) positivity and resilience (8%) – older people presented in a positive light, for their resilience, optimism, gratitude and self-responsibility, (4) older people as grandparents (7%) – described as dear and vulnerable, (5) British economy amid the pandemic (4%) – financial challenges posed by the pandemic, (6) industry dispute in the care sector (3%) – conflicts between private care homes and local authorities over budget and resources, and (7) other (15%).

The thematic categorisation of articles highlighted that older people were depicted mostly in direct relation to risk. I therefore focus on this trend in my analysis. However, to understand how the dominance of risk in older people’s representation during the pandemic compares to their representation in regular times, I ran another search. Using the search terms ‘older people’ and ‘elderly’, I searched for articles published across the same four newspapers that had appeared during each week between 4 February 2019 and 2 August 2019. Following the same procedure of the preceding search, articles from were classified into six categories: (1) risk (41%) – morbidity, illness and poverty among older people, (2) scientific research and innovations (11%) – scientific breakthroughs in geriatric medicine, (3) positivity (10%) – older people and ageing presented in a positive light, (4) charity/community (4%) – social initiatives for older people (e.g. making digital platforms accessible), (5) older people and Brexit (5%) – articles discussing the identification of older people as leavers, and (6) other (27%).

The data reveal that risk constitutes a dominant context in older people’s newspapers representation in both 2019 and 2020, that is, pre and post the outbreak of Covid. Yet, as the different categories of articles published in 2019 and 2020 suggest, there is a particular discursive backdrop against which the notion of risk unfolds in relation to older people within each year. For example, 11 percent of the articles published in 2019 were about scientific research and innovation in relation to old age. These articles approach ageing and old age as amenable processes, where health conditions and problems can be ameliorated with the right medical interventions. The overall tone of the discussions is cautiously promising, and while older people are depicted as subjects of risk, the focus is on treatment and improvement of their health. This stream of articles is informed by discourses of positivity underscoring optimistic views of ageing while promoting neoliberal imperatives of self-responsibility in ageing successfully. Such articles do not appear during 2020, and I suggest that this difference reflects that there are distinct discursive atmospheres within each year that impact the kind of cultural work that risk is doing in relation to the older population.

The process of close and reiterated reading of the appearances of risk across articles has elucidated that the risk of Covid-19 was discussed as being unmanageable in relation to older people. Building on the approach of scholars like Jonathan Potter and Margaret Wetherell (1987), Michael Billig (1991) and Rosalind Gill (2000) to discourse as a social practice, my analysis shares a ‘conviction in the rhetorical organisation of discourse’ (Gill, 2000: 174), and aims to identify the various constructive processes that lie at the heart of discourse. Yet, far from monolithic, the discourse of unmanageable risk appeared to be multifaceted as it evolved throughout three distinct phases. In each phase, I therefore analyse the dominant discursive structure as a variation of the unmanageable risk discourse.

While the findings and following analysis provide a close and detailed account of the main tropes operating within the discourse of unmanageable risk, this research is not exhaustive of the way older people were represented in UK print media during the pandemic. Non-verbal features within articles, for example, images, font or layout were not included in the data, while the focus on ‘risk’ meant that other, less prominent contexts in which older people are mentioned, were left unpacked. In addition, even though the analysis detects the variations of the unmanageable risk discourse, further research is required to go into the depth of why these variations occur.

## Young versus old

The first article mentioning older people in the pandemic, published by the *Telegraph* on 13 February 2020, categorised them alongside children as those being most at risk to contract the virus and die. Yet, while the risk of older people to die from the virus is only merely mentioned (‘British pensioners have certainly come to uncomfortable proximity to the virus’), for the young the tone is alarming. ‘Understandably’, goes the article, ‘there have been reports of young people growing increasingly anxious about the spread of what may seem to them like an apocalyptic disease’ (Shute, 2020).

Taking on various forms, a binary between young and older people had been persistently produced across the news. Indeed, even when more and more evidence had underscored that it is older people who might be more susceptible to Covid-19, the analogy between young and old continued to be cited in many articles. Resonating with the observation made by Carney et al. (2022) that age in the pandemic served as a key factor in determining who dies and who survives, I suggest that the opposition between the young and the old was central in configuring the risk of Covid-19 as unmanageable for older people.

The negation of older people with ‘the young’ on the basis of heightened risk gained currency particularly through the early discourse of ‘protective rings’ and ‘shielding’, in which older people were mentioned in line with people who have underlying health issues, and sometimes also with disabled people.^2^ The consistent lumping together of older people with those dealing with underlying health issues and/or disabilities helped to establish the idea that these populations are more likely to die due to their vulnerable bodies. Moreover, the negation of these marginalised groups with ‘the young’ homogenised them not only through the attribution of an increased susceptibility to Covid-19, but also that of a decreased worth of life. As one *Telegraph* article published in the end of February alerted, ‘The virus is more likely to affect the elderly and those with underlying health conditions. However, a not insignificant number of healthy young people have died too - and this is worrying doctors around the world’ (Nuki, 2020).

Some of these articles, which were published weeks before deaths started to occur, exemplify that through the juxtaposition to younger people, the risk of the virus was configured as imminent for older and vulnerable bodies. This way, the more older people’s injuries were rendered a predictable outcome, the more the risk posed to their lives was ingrained as unmanageable.

The predictability and inevitability of older people’s injuries continued to be ascribed to them through the juxtaposition to younger people. While at times, this juxtaposition served to mitigate public fears (‘So providing you aren’t elderly or suffering from an existing illness, you needn’t panic’),^3^ in other times, it served to amplify those fears. Two examples are an article published by the *Guardian* on 24 March:

At first it was only elderly people. The narrative about coronavirus, fanned by the details of every sad death announced, was that the virus was mainly a concern for those over 70 [. . .] But we’re beginning to see that coronavirus can make some younger people seriously ill. [. . .] As with most things in this pandemic, the idea that coronavirus only threatens older people is an oversimplification. (Ball, 2020)

And this *Telegraph* article published on 27 March:

At the start of the outbreak, messaging was tethered to protecting the elderly and vulnerable. This led to certain demographics failing to adopt behaviours to control community spread. It is no surprise that there has been an uptick in stories about younger victims around the world. Though still a significant minority of cases, heart wrenching examples are being exchanged internationally through global necessity to shift the narrative. (Phillips, 2020)

Both articles exemplify that while the juxtaposition between younger and older people is meant to produce younger lives as more worthy, it also reinforces the view that not a lot can be done to manage the risk in relation to older people. As Mladenov and Brennan (2021) have argued in relation to disabled people, the media’s emphasis on enhanced susceptibility along with the focus on individual vulnerability have steered the attention away from the massive impact of structural inequalities and harmful policy decisions on marginalised populations. Following this, I suggest that through the young versus old binary, not only enhanced susceptibility was ascribed to older people but also unmanageability of their risk. And similar to the media representation of disabled people during the crisis, the young versus old binary worked to divide the body politic to those deserving and those who are less so, and to justify policy decisions and economic conditions set by the neoliberal government.

More specifically, the disposal of older people against the young in this way helped to buttress the underlying paradigm behind the UK government strategy of herd immunity, that Isabel Frey (2020) has termed ‘epidemiological neoliberalism’. Frey cogently highlights how the government’s approach of letting the virus spread follows a clear neoliberal logic: just like the market, the pandemic is best handled when left unregulated. Yet, in line with scholars, such as Rose (1999), Larner (2000), Lemke (2001) and Brown (2003) who already depicted the many institutional and legal frameworks organised by the neoliberal state to enable a market to exist and to provide its needs, I argue that the deregulation of the pandemic was, too, directed through deliberate means, and towards specific goals. The binary of young versus old constitutes a discursive means through which policies led by the neoliberal government to cut down care services and resources for older people during the pandemic were shaped as ethically tolerable. By constructing the risk of older people to suffer from the virus as irreducibly biological and thus inescapable and unmanageable, the young versus old binary emphasised the presumed vulnerability of older people, while framing care for this population as having tenuous prospects to help them survive. In this way, the fact that the transmission of the disease among hundreds of thousands of older people resulted from the steady depletion of resources and the denial of care for this population was blurred and justified at the same time.

## Claps for Tesco

From March to mid-April, issues around food, medications and other supplies for the self-isolating older demographic were discussed extensively and intensively, defining the predicament of older people within the contours of the private and domestic sphere. While the emphasis on supplies helped to deflect attention from public resources to individual ones, it also incited two discourses that, in turn, reinforced the link between older people and unmanageable risk.

First, at the same time as the media incessantly mentioned the heightened risk of older people, the focus on supplies engendered an enthusiastic coverage of the heartening mobilisation of voluntary initiatives for this population. As one *Telegraph* article published on 10 March under the title ‘My coronavirus cure? It’s time to put the grandparents on lockdown’ exemplifies, the mobilisation of charities in addressing the needs of the older population was fervidly discussed:

Who exactly is going to make sure they have everything they need if they can’t leave the house? Perhaps Esther Rantzen’s excellent Silver Line could start taking messages from callers who need supplies and tip off local communities to drop stuff on their doorstep? Over to you, Dame Esther, we’re ready and willing! (Pearson, 2020)

While the article questions the viability of the instruction given to older people to self-isolate, any demands from government or local authorities are deflected and instead hot lines and local communities are galvanised into action with excitement. Another article published by the *Telegraph* on 19 March praised the initiatives of private businesses:

Meanwhile, staff at London-based tour operator Experience Travel Group have offered support to vulnerable people in the local community. Co-founder Sam Clark said: Staff have made contact with their neighbours at home to offer help with shopping, and they’ve volunteered to provide ‘meals on wheels’ to a local lunch club for the elderly that has had to close its doors. We have also circulated information as widely as we can about an online food bank scheme. (Plush, 2020)

The article subsequently depicts how a blood donation drive for elderly people was set by a private business, while utilising a positive and boastful tone. Similar in their rhetoric, articles with titles like ‘A crisis can bring out the best in us: Meet the small businesses creating hope’, and ‘Community spirit makes comeback amid anxieties of lockdown Britain’, emphasised the vast volume and range, as well as endless dedication of volunteers. This celebratory discourse converges with what Lisa McCormick (2020) analysed as a militaristic language within British pandemic discourse. Not unlike the blustery discourse of the disease as a war, the celebration of altruism towards older people operated to induce solidary sentiments while heroising particular social groups.

Increasingly, the positive language heroising volunteers was employed in the articles in a way that eclipsed the unfolding neglect of older people led by the government’s neoliberal policy measures. At the same time, these depictions of volunteerism and kindness helped to shape the care given to older people as a spontaneous, altruistic gesture, rather than an imperative. Under the emphasis of volunteerism then, older people were placed outside the protection of the state, while the care they were reported to have received was articulated more as aid rather than means to avert the risk to which they are exposed.

Intersecting with the celebratory coverage of volunteerism, the second discourse that stemmed from the media’s focus on supplies portrayed the pandemic as a natural force. Thus, the admiration towards the random, makeshift support network for older people not only overshadowed their actual neglect and undermined their entitlement for institutionalised care, but also assisted in framing the crisis as an unruly catastrophe. An article published by the *Daily Mirror* on 16 March under the title ‘Protect the vulnerable’ is one example:

CHARITY bosses and union chiefs have demanded urgent action to keep pensioners supplied with food, medicines and essentials when they go into self-isolation. The call came after Health Secretary Matt Hancock warned millions of people over 70 will have to hide themselves away for four months in a bid to avoid being hit by coronavirus. (Glaze and Martin, 2020)

As exemplified here, while the demand of charities for supplies was possibly critical of the government, in the article this demand nevertheless intensifies descriptions of the pandemic as an uncontrollable event. Phrases portraying older people as having to ‘hide themselves away’, ‘in a bid to avoid being hit’, evoke a sense of unpredictability, emphasising ‘the power of chance over the efficacy of public health prevention efforts’ (Brandt and Botelho, 2020: 1493–1494). Similar language was employed by the MP chairing the Ageing and Older People All-Party Parliamentary Group quoted in the article saying that she ‘hopes an “army of volunteers” will step up to support efforts [. . .] before the storm comes’ (Glaze and Martin, 2020). I suggest that such instructions for older people to disappear to avoid death reflect both how they were held responsible for their own risk (Carney et al., 2022) and that a sense of fatalism was entrenched in relation to their survival.

Crucially, while their predicament was both eclipsed and subjected to an uncontrollable danger, older people themselves were invisibilised through and through, as their names, voices and testimonies were consistently absent from the articles. Instead, with care rendered voluntary and risk unmanageable, older people were homogenised and produced as an unprotectable and invisible mass, while the inadequacy of the care system was disavowed.

By the end of April 2020, 29,561 people aged 65 and over had died due to the virus, constituting 88 percent of the dead. Yet, the fact that they were dying *en masse* was neither acknowledged nor dramatised before mortality statistics in care homes began to be included within the official death toll published in the media on 20 April. Once these mortality statistics were avowed, the mass deaths in care homes captured most of the focus of the media’s coverage of older people through April and May 2020.

## Dying anonymously: the care homes debacle

The bombast, militaristic discourse celebrating the altruism of the British public has shifted in the month between mid-April to mid-May as numerous articles began publishing alarming depictions of the calamity occurring in care homes. In stark contrast to the boastful tone in the previous period, news stories started exposing the systematic breakdown of the privatised care homes system while outlining the extent of the continued neglect of the older population. Depicting the grim reality of a collapsing care system, articles published during this period pronounce a clear tone of horror against the suffering of care home residents.

However, even as the tone of the news had shifted and dramatised the plight of this population, possibly pressuring the government to direct more resources and delay the spread of the virus across care homes (Daly, 2020: 990), it did not shift the way in which older people themselves and the risk of the virus to their lives were represented. First, while articles display an affective register of shock, sadness and even anger against the abandonment of older people, care home residents themselves remain invisible and voiceless. In fact, the visualisation of the horror in care homes worked in tandem with the erasure of older people themselves, demonstrating how the avowal of older people’s suffering was tied to their disavowal as real individuals. Second, while older people were erased, the risk posed to their lives was reiterated as being devastating and yet unmanageable. One of the early articles reporting on the spread of the virus across care homes, published by the *Telegraph* on 14 April, is a paradigmatic example of this discourse:

Manager Rachel Beckett, of Wellburn Care Homes, near York, said she was forced to play ‘Russian roulette’ with her residents because of a lack of testing of discharged patients. ‘The instruction from Public Health England said that “because of the lack of testing available, the readmissions may or may not have Covid-19”’, she said. “To expect us to comply with these instructions is tantamount to playing Russian roulette with the lives of our most vulnerable, the very people we’re here to keep safe and protect”. (Knapton, 2020)

Even as residents are mentioned in the article as the main concern of the workers taking care of them, it is the experience of care workers that constitutes the main subject. Narratives of care home residents themselves are absent, as residents are backgrounded as helpless candidates for a dreadful fate. Rather than residents themselves, it is the Russian roulette metaphor that is positioned at the fore of manager Beckett’s account. What is emphasised through the metaphor – which is stated twice in the article – is the random culling of older people, while the referents remain voiceless and deprived of agency. Thus, inadvertently, this metaphor visualises the horror of their state while dispossessing older individuals of what makes them acknowledgeable: their names or personal histories are absent and instead what is prioritised is their predicament. Similar to people with dementia who are often depicted as ‘merely the space upon which dementia advances and pharmaceutical drug resist’ (Bailey et al., 2021), older people were portrayed – in this article as well as many others – mainly as targets of the virus.

Furthermore, working in conjunction with this dispossession, the Russian roulette metaphor emphasises older people’s risk of getting hit by the virus as spiralling out of control. While the framing of an arbitrary death machine may be alarming, it also paradoxically reinforces a fatalist sense of a catastrophe that can no longer be managed. Such framing is coupled with the construction of older people as an unseen and unheard crowd, in a way that contributes to a kind of public senselessness towards the loss of tens of thousands of older people. In other words, even as their plight is depicted with urgency, since this plight bears no voice, face or name, it soon sinks back into the margins of the popular imagination.

Similar metaphors that anonymise older people while construing their risk as unmanageable continued to appear in subsequent articles. For example, a *Telegraph* article from 15 April that focused on care home providers’ experience depicted Covid-19 as spreading across anonymous older residents ‘like wildfire’ (Gardner, 2020). Another article published by the *Guardian* on 29 April portrayed older people merely as being slayed by the virus, like in events of massive destruction:

There are undoubtedly people in the UK who ignore guidance. Not many, but some. A person like that could ultimately be responsible for going into a care home and introducing the infection, causing 20–30 residents to die over the next month. (Mason et al., 2020)

The discourse employed here emphasises the scale of destruction, while anonymising the victims of this destruction, or describing them solely in terms of their quantity and mortality. Whether it is the Russian roulette metaphor, or that of a wildfire, the simultaneous visualisation of the virus and invisibilisation of older residents brought critical moments within older people’s state of emergency to be shaped in a way that sustained an already existing sense of despair in relation to them.

Notably, this discourse was fostered by a particular temporal register of the events as transpiring in real time. In several articles, metaphors that were explicitly temporal were used, like in this *Telegraph* article from 15 April:

Jeremy Hilton, the group leader on Gloucester City Council, said a care home under his supervision had been hit by a major outbreak after accepting around six hospital patients suffering from Covid-19. ‘A person is dying at that home every day now’, he told The Telegraph (Gardner, 2020)

The depiction of the disaster in care homes in present progressive tense dramatises the emergency at the same time as it reinforces the sense that the disaster is unfolding at an unbeatable pace. Thus, while many called for immediate action, given the erasure of older people and the construal of their risk as unmanageable, the discourse articles employed often incited inaction. Amplified by the metaphors as occurring in real time, horror was prioritised in the articles over the depiction of real older people whose lives may have been hurt. Instead of attending to testimonies of individuals, the focus was on the crisis as exceeding the possibility of control. Another example of this can be seen in an article published by the *Daily Mail* on 18 May:

Lawyers, care providers and charities say they have been ‘inundated’ with calls from families wanting to remove relatives from homes over fears the facilities are ‘ticking time bombs’.

[. . .] Emma Jones, a human rights solicitor at Leigh Day, said before coronavirus it was ‘highly unusual’ to be approached by clients wanting to move relatives out of care homes. ‘Now I’m in the position where there are a number of people contacting me every week’, she said. Miss Jones added: ‘There is fear about hospital discharges, the lack of PPE equipment, [families] can’t even visit them. It’s easy to understand how people see this as a ticking time bomb’. (Coen, 2020)

The metaphor of a ticking time bomb maintains the duality of urgency and predetermination. Like other metaphors, the ticking time bomb metaphor dramatises the collapse itself, pronounced in the increasing phone calls to remove relatives from care homes, as well as through the accumulation of failures. Yet, while an urgent call for action may be implicit in the article, what is resonated through the ticking time bomb metaphor is the image of older people in care homes as already dying and the prospect of survival as tenuous. The coupling of older people’s portrayal as an anonymous mass with the construal of their lives as lost, helped to limit the discourse from avowing older people as real individuals whose lives and experiences are worthy of public attention.

## Conclusion

Taking various forms throughout the pandemic, the discourse of unmanageable risk produced the idea that managing the risk of older people to contract and die from Covid-19 is a tenuous endeavour. By constructing their risk as unmanageable, this mechanism of governance helped to position older people outside of the protection of the state. As my analysis demonstrates, central to the construction of unmanageable risk was the invisibilisation and anonymisation of older people by this discourse that simultaneously depicted them as frail and unprotectable. This bleak depiction, however, seems to be in contrast with the ever more positive visibility of older people, that has become dominant in mainstream media in the past decade.

Indeed, the outbreak of Covid-19 occurred during a historic moment in which older people (and particularly older women) have become much more visible across mainstream media and popular culture (Dolan, 2013; Jermyn 2021; Jermyn and Holmes 2015). Not only have they become more visible but, as I demonstrate elsewhere (Shimoni, 2018, 2023), in the past decade older subjects have been increasingly interpellated as self-reliant entrepreneurs, who, upon making ‘right’ self-investments, are able to age successfully and remain healthy, resilient and happy. Such positive portrayal, in turn, has helped to conceal the erosion of social welfare by neoliberal policies, and its impact on the vast majority of older people.

Thus, the emergence of unmanageable risk discourse reflects how the media coverage of older people has shifted focus in the pandemic, emphasising not the good health and proactive spirit of those aged 65 and over, but the intensely vulnerable and helpless state of this population. I therefore conclude by asking: considering the stark cultural contrast to their positive portrayal pre-Covid, how should we understand the emergence of the unmanageable discourse with its overt presentation of older people as frail and unprotectable? Does this discourse disrupt the neoliberal interpellation of older subjects as resilient and self-sufficient?

Chris Gilleard’s and Paul Higgs’ (2010) theorisation of the fourth age provides important insight into whether the negative pandemic portrayal of older people undermines their cultural image as normative neoliberal subjects who are lively and agentic. Gilleard and Higgs (2010) go beyond Peter Laslett’s famous definition of the fourth age as a phase of dependence, decrepitude and death, and underscore the cultural role of the fourth age as a metaphorical ‘black hole’ of ageing that represents the ‘shadowlands of disability, diminishment and death’ (p. 126). They argue that while the fourth age exercises a powerful impact on the cultures of ageing, it does not represent a particular age group or a stage of life, but a state of ‘unbecoming’ (Higgs and Gilleard, 2014: 13).

I suggest that similar to the fourth age, the unmanageable risk discourse exercised its power by denoting a horrid prospect for older people who were stripped from any agency and identity. Building on Gilleard and Higgs, it is precisely the anonymity and invisibility of the unprotectable older subject that enabled unmanageable risk to effectively promote the portrayal of the older demographic as disposable, without risking the image of the older subject as happy and agentic. Thus, the absence of older people’s names, gender and voices from articles not only delimited what *can* be represented, seen, felt or known about their experiences and views (Butler, 2004: XX), but by constituting an abject, unspecified ‘other’, it ensured the entrenchment of neoliberal governmentality amid a crisis.

## Notes

1. According to newsworks’ website, while the *Guardian* has a monthly reach of more than 23 million (https://www.newsworks.org.uk/the-guardian) and the *Telegraph* stands with more than 18 million (https://www.newsworks.org.uk/the-daily-telegraph), both the *Daily Mail* (https://www.newsworks.org.uk/daily-mail) and the *Daily Mirror* (https://www.newsworks.org.uk/daily-mirror) have more than 30 million readers per month.

2. As the data reveal, people with disabilities were seldom mentioned in articles where older people were discussed. More often articles used the wide notion of ‘the vulnerable’, sometimes breaking this category down to specific chronic medical problems, but not disabilities. I suggest that this omission reflects both how the intersection of age with forms of disability was ignored (Goodley et al., 2022), and that disabled people are placed in a separate category within the social margins, that of the abnormal (see Davis, 2021).

3. Chandler-Wilde et al. (2020).
